# Effects of *Fagopyrum dibotrys* rhizoma meal supplementation on productive performance, egg quality, egg nutritional value, and serum biochemical parameters of *Shanma* laying ducks

**DOI:** 10.3389/fvets.2025.1654416

**Published:** 2025-08-06

**Authors:** Pingwen Xiong, Gaoxiang Ai, Jiang Chen, Wenjing Song, Weide Su, Dongyou Yu, Qiongli Song, Chuanhui Xu, Zhiheng Zou, Qipeng Wei, Xiaolian Chen, Lizhen Hu

**Affiliations:** ^1^Key Laboratory of Molecular Animal Nutrition (Zhejiang University), Ministry of Education, Hangzhou, China; ^2^Institute of Animal Husbandry and Veterinary Science, Jiangxi Academy of Agricultural Sciences, Nanchang, China; ^3^Jiangxi Province Key Laboratory of Animal Green and Healthy Breeding, Nanchang, China

**Keywords:** *Fagopyrum dibotrys* rhizoma meal, productive performance, egg quality, egg nutritional value, serum biochemical parameters, *Shanma* laying ducks

## Abstract

**Introduction:**

The rhizoma of *Fagopyrum dibotrys* (D. Don) Hara, a traditional natural medicinal herb with extensive historical applications in China, possess anti-inflammatory, anticancer, antioxidant, antimicrobial, immunomodulatory, and antidiabetic effects. However, the potential positive effects of *F. dibotrys* rhizoma meal (FDRM) on productive performance in high-density laying duck farming remain unclear. This experiment was conducted to assess the impacts of FDRM supplementation in *Shanma* laying ducks diet by determining productive performance, egg quality, egg nutritional value, and serum biochemical parameters.

**Methods:**

With similar laying performance (80.88 ± 5.17%) and body weight (1.24 ± 0.02 kg), 512 healthy 32-week-old *Shanma* laying ducks were randomly assigned to four groups consisting of eight replicates (16 ducks per replicate). Ducks in the control group (F0 group) were fed only the basal diet, while the other groups (F1, F2, and F3 groups) were fed the basal diets supplemented with 1, 2, and 3% FDRM, respectively. The experiment lasted for 49 days with *ad libitum* access to feed and water.

**Results:**

The results showed that supplementing FDRM in duck diet had no adverse effects on laying performance (*p* > 0.05). Additionally, compared with the control group, dietary supplementation with FDRM significantly improved the shell strength, yolk color, and shell proportion (*p* < 0.05), while increasing the serum total protein (TP) content (*p* < 0.05). The study also found that adding 2% FDRM significantly enhanced the contents of total amino acids, essential amino acids, and umami amino acids in eggs (*p* < 0.05), improved the composition of monounsaturated fatty acids and polyunsaturated fatty acids (*p* < 0.05), and reduced the saturated fatty acids content. However, 3% FDRM addition increased the serum blood urea nitrogen content (*p* < 0.05), indicating reduced the dietary protein utilization efficiency.

**Discussion:**

With the rapid development of the economy and the continuous improvement of people’s living standards, people have raised higher demands for the nutritional and high quality of eggs. Duck eggs, rich in protein, amino acids, fatty acids, minerals, and vitamins, serve as an important source of high-quality protein for human’s food and health. Moreover, the n-3 polyunsaturated fatty acids in eggs have beneficial effects in preventing cardiovascular diseases. Currently, numerous studies have shown that *F. dibotrys* is abundant in active substances such as flavonoids and phenolics. Additionally, Traditional Chinese herbs rich in flavonoids and phenolics have been proven to enhance the nutritional value of eggs, improve the laying performance of poultry, and promote their overall health. This study indicated that dietary supplementation with 2% FDRM might improve egg quality and egg nutritional value of *Shanma* laying ducks through improving the shell strength, yolk color, and shell proportion, enhancing yolk fatty acids and amino acids profiles and elevating serum TP content.

## 1 Introduction

China is the world’s largest producer of laying ducks, maintaining a standing stock of approximately 150 million ducks annually, which accounts for over 80% of the global total (1). Amid the rapid development of the laying duck industry, key industry concerns now revolve around enhancing production performance, optimizing egg quality, improving the nutritional value of duck eggs, and achieving green and sustainable development. Additionally, duck eggs are rich in protein, amino acids, fatty acids, minerals, and vitamins, serving as an important source of high-quality protein for humans (2, 3). The fatty acids in eggs are also beneficial to human health, particularly the n-3 polyunsaturated fatty acids in eggs, which have a beneficial effect on preventing cardiovascular diseases (4). With rapid economic development and continuous improvement in living standards, consumers are placing higher demands on the nutritional and health-promoting qualities of egg products, making it imperative to enhance the nutritional and functional value of duck eggs (3). Recent studies have revealed that Chinese herbal medicine or plant extracts are rich in bioactive compounds such as disease-resistant alkaloids, antioxidant flavonoids, and phenolics. These substances can effectively improve the nutritional value of eggs by enhancing antioxidant capacity, boosting immune function, and optimizing gut microbiota structure in poultry, demonstrating significant potential for practical applications (5–12).

*Fagopyrum dibotrys* (*F. dibotrys*) (D. Don) Hara, a perennial herbaceous plant belonging to the *Polygonaceae* family and *Fagopyrum* genus, possesses significant medicinal and edible value, and has been officially included in China’s Feed Materials Directory (13). The rhizomata, stems, leaves, flowers, and other parts of it contain trace mineral elements, including copper (Cu), iron (Fe), and zinc (Zn), etc., as well as 17 amino acids such as methionine (Met), arginine (Arg), and lysine (Lys), etc., along with various vitamins including vitamin B₁, vitamin B₂, and vitamin E, etc. (14), and these essential nutrients potentially enhancing egg production, improving egg quality, boosting immune function, and supporting overall health and productivity. The rhizoma of *F. dibotrys* (FDR) has a long history of application in traditional Chinese medicine. It contains various bioactive compounds including flavonoids, phenolics, triterpenoids, and tannins, etc. (15–17), and exhibits multiple biological properties such as antioxidant, antibacterial, anti-inflammatory, and immunomodulatory effects (18–20). Currently, its stems and leaves are widely used in pharmaceuticals, health foods, beverages, and forage, while research on its rhizomata in livestock production remains limited. As a forage crop, *F. dibotrys* exhibits strong adaptability, high propagation efficiency, and substantial biomass yield (up to 112,500 kg hm^−2^). Its stems and leaves are rich in crude protein (12.28% DM) with low crude fiber (24.06% DM), neutral detergent fiber (39.74% DM), and acid detergent fiber (30.18% DM), making it a potential unconventional feed ingredient to replace part of swine diets and alleviate feed shortages (14, 21–23). Studies showed that supplementing 10% fresh *F. dibotrys* in laying hens diets deepened yolk color and improved amino acids composition in Changshun green-shell eggs (23). Furthermore, adding 400–800 mg/kg of *F. dibotrys* stem-leaf extract not only enhanced egg production, quality, and nutritional value but also improved immunity by modulating serum biochemical parameters (24). Additionally, dietary inclusion of 1–2% *F. dibotrys* rhizoma meal (FDRM) in broilers alleviated oxidative stress induced by oxidized oil, thereby improving poultry health (19).

Nevertheless, based on our knowledge, researches on *F. dibotrys* has primarily focused on its stems and leaves, with studies conducted on growing-finishing pigs (12), sows (22), mice (20), and laying hens (23, 24), demonstrating certain improvements in animal growth performance and farming efficiency. However, studies on the application of its rhizomata in poultry are very scarce. It is currently unclear whether dietary supplementation with FDRM can exert similarly positive effects on intensive laying ducks production as its stem and leaf derivatives. Consequently, this study aims to investigate the effects of FDRM on productive performance, egg quality, nutritional value of eggs, and serum biochemical parameters, thereby providing a scientific basis for utilizing FDRM as an unconventional feed resource in *Shanma* laying ducks production.

## 2 Materials and methods

### 2.1 Experimental materials

The *F. dibotrys* rhizoma meal (FDRM) used in this study was provided by the Institute of Animal Husbandry and Veterinary Medicine, Jiangxi Academy of Agricultural Sciences. Fresh *F. dibotrys* rhizomata were collected, crushed and passed through the 80-mesh screen to prepare FDRM. The main bioactive compounds of FDRM are total flavonoids and polyphenols, quantified using a UV spectrophotometer (UV-1800, Shimadzu Corp., Kyoto, Japan) at the Jiangxi Province Key Laboratory of Animal Green and Healthy Breeding, which the contents are 19.6 mg/g and 63.85 mg/g, respectively. Moreover, the nutritional compositions of FDRM in this experiment are shown in Table 1. The contents of conventional nutrients in *F. dibotrys* rhizoma meal were determined according to the methods specified in the National Standards of the People’s Republic of China, including gross energy (GE, GB/T 45104-2024), dry matter (DM, GB/T 6435-2014), crude protein (CP, GB/T 6432-2018), crude fat (EE, GB/T 6433-2006), crude fiber (CF, GB/T 6434-2006), crude ash (Ash, GB/T 6438-2007), calcium (Ca, GB/T 6436-2018), and total phosphorus (TP, GB/T 6437-2018).

**Table 1:** Nutritional compositions of FDRM (air-dry basis) %.

Items	Contents
Gross energy (MJ/kg)	15.48
Dry matter	86.40
Crude protein	4.03
Crude fat	0.30
Crude fiber	14.10
Crude ash	4.10
Calcium	0.41
Total phosphorus	0.31

FDRM, *Fagopyrum dibotrys* rhizoma meal.

### 2.2 Ducks, experimental design, and treatments

This study was conducted on 32-weeks-old Longyan *Shanma* laying ducks for a 49-day period with a completely randomized design. A total of 512 laying ducks with similar productive performance (80.88 ± 5.17%) and body weight (1.24 ± 0.02 kg) were used in this experiment. Ducks were randomly allocated to four groups with eight replicates per group and 16 ducks per replicate (128 laying ducks per group). The control group (F0) received the basal diet, while the treatment groups were provided with diets containing 1% (F1), 2% (F2), and 3% (F3) FDRM supplementation. All diets were nutritionally balanced and formulated to meet identical nutritional specifications.

### 2.3 Diets and management

This trial was carried out at the test field of laying ducks in Gaoan, Institute of Animal Husbandry and Veterinary Science, Jiangxi Academy of Agricultural Sciences, PR China. The basal diet fed animals was maize-soybean meal diet, which was formulated based on the China’s national standard “nutrient requirements for egg duck” (GB/T 41189-2021) to meet the nutrient requirements of Longyan *Shanma* ducks. Table 2 presents the composition and nutrient levels of experimental diets. The experimental laying ducks were raised in three-layer three-dimensional netting, consisted of four adjacent cages (40 × 38 × 38 cm, length × width × height) with two animals per cage, providing 28,880 cm^3^ per animal in closed fully automated duck house. Each replicate was raised on the upper and middle floors and each group was guaranteed to be equal in the number of distributed upper and middle layers. During the period of study, the housing temperature and relative humidity were 23.0 ± 2.0°C and 55–75%, respectively. Furthermore, the photoperiod was set at 16L:8D with a light intensity of 20 lux through a 49-day experimental period. Animals were kept with *ad libitum* access to feed and water during the entire experimental period.

**Table 2:** Composition and nutrient levels of experimental diets (air-dry basis) %.

Items	F0	F1	F2	F3
Ingredient				
Maize	52.00	51.60	51.40	51.00
Soybean meal	23.90	23.81	23.75	23.65
Wheat bran	9.35	9.85	10.10	10.60
Soybean oil	1.00	1.00	1.00	1.00
Limestone	8.47	8.46	8.47	8.50
Calcium hydrogen phosphate dihydrate	1.39	1.39	1.39	1.36
Sodium chloride	0.30	0.30	0.30	0.30
Choline chloride	0.15	0.15	0.15	0.15
Vitamin premix^1^	0.03	0.03	0.03	0.03
Mineral premix^2^	0.20	0.20	0.20	0.20
dl-methionine	0.15	0.16	0.16	0.16
l-lysine HCl	0.04	0.04	0.04	0.04
l-tryptophan	0.02	0.01	0.01	0.01
Rice bran and hull	3.00	2.00	1.00	0.00
*Fagopyrum dibotrys* rhizoma meal	0.00	1.00	2.00	3.00
Total	100	100	100	100
Nutrient levels^3^				
Metabolizable energy (MJ/kg)	10.47	10.47	10.48	10.47
Crude protein (%)	16.50	16.50	16.50	16.50
Ca (%)	3.60	3.60	3.60	3.60
NPP (%)	0.35	0.35	0.35	0.35
Digestible lysine (%)	0.854	0.853	0.853	0.852
Digestible methionine (%)	0.399	0.408	0.408	0.408
Digestible threonine (%)	0.210	0.200	0.200	0.201
Digestible tryptophan (%)	0.605	0.605	0.605	0.604

^1^The vitamin premix provided the following per kg of diets: Vitamin A 7500 IU, Vitamin D3 2,500 IU, Vitamin E 20 IU, Vitamin K3 2.5 mg, Vitamin B1 3 mg, Vitamin B2 6 mg, pantothenic acid 20 mg, pyridoxine 2.5 mg, nicotinic acid 27 mg, biotin 0.2 mg, and folic acid 1 mg.

^2^The mineral premix provided the following per kg of diets: Cu (as copper sulfate) 20 mg, Fe (as ferrous sulfate) 50 mg, Zn (as zinc sulfate) 70 mg, Mn (as manganese sulfate) 70 mg, I (as potassium iodide) 0.4 mg, and Se (as sodium selenite) 0.3 mg.

^3^All nutritional levels were calculated based on Tables of Feed Composition and Nutritional Value in China (34th Edition, 2023). FDRM, *Fagopyrum dibotrys* rhizoma meal; F0, control group; F1, the group supplemented with 1% FDRM; F2, the group supplemented with 2% FDRM; F3, the group supplemented with 3% FDRM.

### 2.4 Productive performance

Throughout the trial, the ducks’ egg production and egg weight were monitored daily, and feed consumption was meticulously recorded on a replicate basis at weekly intervals using an electronic balance (HLD-5003, Youheng Weighing Equipment Co., Ltd., Hangzhou, China). At the end of the feeding trial, these values processed using Excel 2016 (Microsoft Corp., United States) were used to analyze the daily egg weight (DEW), average egg weight (AEW), laying rate (LR), average daily feed intake (ADFI), and the ratio of feed to egg (F/E) of the ducks for the 49-day feeding period.

### 2.5 Egg quality

On the final day of the experiment, a random subset of eight freshly laid eggs (a total of 64 eggs from each treatment) were collected for each replicate, which were used for conventional egg quality analysis (within 48 h after laying), including shape index, shell strength, shell thickness, Haugh unit, yolk color, vitellus proportion, albumen proportion, and shell proportion. The shape index was measured with a precision caliper marked at 0.01 mm intervals and was represented by the formula shape index (SI) = (egg length/egg width) (3). Shell strength was assessed along the vertical axis with a compression tester (EFG-0503, Robotmation, Tokyo, Japan). The shell thickness was determined (excluding shell membrane) using a micrometer with the least count of 0.01 mm and was expressed by the mean of measurements taken at three points (air cell, equator, and sharp end) of the egg. Haugh unit, yolk color, and albumen height were measured using an Egg Multi-tester (EMT-5200, Robotmation, Tokyo, Japan). The vitellus, albumen, and shell were separated, weighed, and expressed as a percentage of total egg weight.

### 2.6 Egg nutritional value

After measuring the egg physical parameters, the vitelluses were sampled, lyophilized, and subsequently analyzed for nutrient composition, amino acids composition and fatty acids profile. Nutrient composition included moisture, crude protein (CP), crude fat, cholesterol, and Ca were determined in accordance with AOAC methods (25). Crude protein content was estimated by measuring nitrogen content (Kjeldahl method) with an automatic Kjeldahl nitrogen analyzer (SKD-200, Shanghai Peiou Analysis Instruments Co., Ltd., Shanghai, China) and applying a 6.25 conversion factor. Ether extract was measured using the Soxhlet method with petroleum ether extraction in a Hanon Automatic Soxhlet Extractor (SZF-06A, Shanghai Lichen Instruments Technology Co., Ltd., Shanghai, China). The method for the Ca and cholesterol determination were used by an UV spectrophotometer (UV-1800, Shimadzu Corp., Kyoto, Japan) and commercial kits (Nanjing Jiancheng Bioengineering Institute, Nanjing, China) as described by Zhang et al. (26).

According to the methods reported by Cullere et al. (27) and Xu et al. (28), the amino acids content of the eggs were analyzed. The determination of amino acids content in egg yolk was determined by ion-exchange chromatography with the following procedure: approximately 0.1 g of egg yolk powder sample was weighed and digested with 5 mL of 6 mol/L HCl solution at 105°C in an oven for 24 h. After digestion, the solution was diluted to 50 mL in a volumetric flask with deionized water and filtered through a 0.22 μm aqueous-phase filter into a centrifuge tube. Then, 2 mL of the filtrate was evaporated in an evaporating dish in a 60°C water bath, followed by the addition of 4 mL of 0.02 mol/L HCl solution for dissolution. Once fully dissolved, the sample was stored at 4°C for analysis using an ion-exchange amino acid analyzer (L8900, Hitachi, Tokyo, Japan). A total of 17 amino acids were determined, as detailed in Table 3.

**Table 3:** Effect of FDRM on amino acids composition in the vitellus of laying ducks^1^ (mg/g, as-fresh basis).

Items^4^	Groups				SEM^2^	*p*-value^3^
	F0	F1	F2	F3		
EAAs						
Thr	6.25^b^	6.38^ab^	6.77^a^	6.82^a^	0.07	0.003
Val	8.09^ab^	8.01^b^	8.46^a^	8.11^ab^	0.09	0.002
Met	4.00	4.31	4.52	4.42	0.08	0.104
Ile	6.14^ab^	6.04^b^	6.56^a^	6.20^ab^	0.08	0.024
Leu	12.26^b^	12.89^b^	13.67^a^	12.95^ab^	0.19	0.004
Phe	6.56	6.46	7.04	6.67	0.10	0.195
Lys	10.10	10.52	11.00	10.84	0.13	0.073
NEAAs						
Asp	12.24^b^	12.42^ab^	13.29^a^	13.26^a^	0.15	0.009
Ser	11.29	11.03	12.08	11.35	0.14	0.066
Glu	17.44^b^	17.77^ab^	18.84^a^	18.28^ab^	0.18	0.026
Gly	4.29^b^	4.35^b^	4.69^a^	4.47^ab^	0.05	0.010
Ala	7.14	7.27	7.42	7.44	0.06	0.307
Cys	2.18^b^	2.20^b^	2.28^a^	2.26^ab^	0.02	0.003
Tyr	5.15	5.36	5.64	5.50	0.08	0.227
His	3.18	3.30	3.42	3.44	0.04	0.092
Arg	8.68	9.07	9.85	9.48	0.17	0.227
Pro	4.96	4.80	5.29	4.84	0.07	0.095
Total EAAs	53.73^b^	54.99^b^	58.75^a^	54.80^ab^	0.66	<0.001
Total NEAAs	74.39	77.08	79.13	79.80	0.97	0.198
Total AAs	128.12^b^	131.61^ab^	140.00^a^	134.60^ab^	1.34	0.008
Flavor AAs	52.46	53.62	55.49	55.61	0.57	0.158
Umami AAs	29.67^b^	30.19^ab^	31.93^a^	31.54^ab^	0.31	0.021
Sweet AAs	27.98	27.45	28.01	28.10	0.38	0.939
Aromatic AAs	11.37	11.82	12.63	12.17	0.17	0.054

^1^Data are the mean of eight replicates with 16 ducks each.

^2^SEM (standard error of the mean): the standard error of the average.

^3^In the same row, values with no letter superscripts mean no significant difference (*p* > 0.05), while with different letter superscripts mean significant difference (*p* < 0.05).

^4^Asp, asparc acid; Thr, threonine; Ser, serine; Glu, glutamic acid; Gly, glycine; Ala, alanine; Cys, cysteine; Val, valine; Met, methionine; Ile, isoleucine; Leu, leucine; Tyr, tyrosine; Phe, phenylalanine; Lys, lysine; His, histidine; Arg, arginine; Pro, proline; EAAs, essential amino acids; NEAAs, non-essential amino acids; AAs, amino acids; Flavor AAs = Asp + Glu + Gly + Ala + Tyr + Phe; Umami AAs = Asp + Glu; Sweet AAs = Ser + Gly + Ala + Pro; Aromatic AAs = Tyr + Phe. FDRM, *Fagopyrum dibotrys* rhizoma meal; F0, control group; F1, the group supplemented with 1% FDRM; F2, the group supplemented with 2% FDRM; F3, the group supplemented with 3% FDRM.

The freeze-dried egg yolk samples were pulverized and passed through a 40-mesh sieve for fatty acids analysis. The preparation of fatty acids methyl esters from total lipids followed the procedure described by Hao et al. (29) and GB 5009.168-2016. Both quantitative and qualitative analyses were performed using an Agilent 7890B gas chromatography system (Agilent Technologies, Santa Clara, California, United States) coupled with an Agilent 5977B mass spectrometer (Agilent Technologies, Santa Clara, California, United States). First, the total lipids of egg yolk samples were extracted using a mixture of chloroform and methanol (2,1, v/v). The lipids were then methylated in a potassium hydroxide-methanol solution (0.4 mol/L) for 30 min, followed by the addition of 2 mL of deionized water. The mixture was vortexed and centrifuged for 5 min (1,006 × *g*), and the upper layer was collected and stored at −20°C for further use. The fatty acids in the samples were identified by combining retention times and mass spectral characteristics. Each fatty acid was identified by comparison with known standards (Anpel Laboratory Technologies Inc., Shanghai, China), and the fatty acids content were calculated using the area normalization method, expressed as a percentage of the total fatty acids. A total of 15 fatty acids were determined, as detailed in Table 4.

**Table 4:** Effect of FDRM on fatty acids profile in the vitellus of laying ducks^1^ (%).

Items^4^	Groups				SEM^2^	*p*-value^3^
	F0	F1	F2	F3		
C14:0	0.40^a^	0.39^ab^	0.36^b^	0.34^b^	0.01	<0.001
C16:0	21.82^a^	21.19^b^	21.15^b^	21.44^ab^	0.08	0.034
C17:0	1.21	1.18	1.15	1.17	0.01	0.081
C18:0	6.64	6.62	6.67	6.64	0.02	0.884
C20:0	0.07	0.08	0.07	0.07	0.001	0.111
C16:1	4.65	4.73	4.75	4.72	0.02	0.116
C17:1	1.32	1.37	1.29	1.30	0.01	0.323
C18:1n9	45.70	45.88	46.01	46.34	0.10	0.106
C20:1	0.32^b^	0.33^ab^	0.33^ab^	0.34^a^	0.003	0.007
C18:2n6	15.20^ab^	15.42^a^	15.33^ab^	14.81^b^	0.09	0.033
C18:3n3	0.12	0.12	0.13	0.13	0.001	0.907
C18:3n6	0.90	0.91	0.91	0.92	0.003	0.082
C20:3	0.085^b^	0.089^ab^	0.087^ab^	0.092^a^	0.001	0.025
C20:4	0.93	0.95	0.93	0.94	0.004	0.197
C22:6	0.75	0.74	0.74	0.74	0.002	0.053
Total SFAs	30.31^a^	29.46^b^	29.41^b^	29.67^b^	0.10	0.033
Total MUFAs	51.98	52.31	52.40	52.71	0.11	0.204
Total PUFAs	17.96^ab^	18.23^a^	18.11^ab^	17.62^b^	0.08	0.041
Total UFAs	69.70	70.55	70.55	70.33	0.11	0.069
UFAs:SFAs	2.30^b^	2.40^a^	2.39^a^	2.37^ab^	0.01	0.040
PUFAs:SFAs	0.59^b^	0.62^a^	0.61^ab^	0.60^ab^	0.005	0.032

^1^Data are the mean of eight replicates with 16 ducks each.

^2^SEM (standard error of the mean): the standard error of the average.

^3^In the same row, values with no letter superscripts mean no significant difference (*p* > 0.05), while with different letter superscripts mean significant difference (*p* < 0.05).

^4^SFAs = C14:0 + C16:0 + C17:0 + C18:0 + C20:0; MUFAs = C16:1 + C17:1 + C18:1n9 + C20:1; PUFAs = C18:2n6 + C18:3n3 + C18:3n6 + C20:3 + C20:4 + C22:6; UFAs = MUFAs + PUFAs; SFAs, saturated fatty acids; MUFA, monounsaturated fatty acids; PUFAs, polyunsaturated fatty acids; UFAs, unsaturated fatty acids; FDRM, *Fagopyrum dibotrys* rhizoma meal; F0, control group; F1, the group supplemented with 1% FDRM; F2, the group supplemented with 2% FDRM; F3, the group supplemented with 3% FDRM.

### 2.7 Serum biochemical parameters

At the end of the 8 weeks, two laying ducks with close to the average weight were randomly selected from each replicate, after fasting, 5 mL of blood samples was collected from the wing vein using a black 7-gauge needle and vacuum coagulation tubes. The blood samples were centrifuged at 1,006 × *g* for 10 min to separate serum within 2 h after blood collection using a centrifuge (LC-LX-L50C, Shanghai LiChen Instrument Technology, Ltd., Shanghai, China) at the Jiangxi Province Key Laboratory of Animal Green and Healthy Breeding, and the serum was separated and stored at −20°C for further use. The serum levels of triglycerides (TG, Cat no, A110-2-1), total cholesterol (TC, Cat no, A111-2-1), high-density lipoprotein (HDL, Cat no, A112-2-1), and low-density lipoprotein (LDL, Cat no, A113-2-1), total protein (TP, Cat no, A045-2-2), albumin (ALB, Cat no, A028-2-1), blood urea nitrogen (BUN, Cat no, C013-2-1), alkaline phosphatase (AKP, Cat no, A059-2-2), and calcium (Ca, Cat no, C004-2-1) were measured by an Automatic Biochemistry Instrument (BS-420, Shenzhen Myriad Bio-Medical Electronics Co., Ltd., Shenzhen, China) using commercial assay kits. All assay kits were provided by the Nanjing Jiancheng Bioengineering Institute.

### 2.8 Statistical analyses

All data were organized using Excel 2013, subjected to tests for normality and homogeneity of variance, and subsequently analyzed using one-way analysis of variance (one-way ANOVA) with the Bonferroni method in SPSS 22.0 statistical software (SPSS Inc., Chicago, IL, United States) to test for multiple comparisons. The experimental results were presented as mean and pooled SEM. A value of *p* < 0.05 was considered statistically significant, while a value of 0.05 < *p* < 0.10 indicated a trend toward an increase or decrease.

## 3 Results

### 3.1 Productive performance

The effects of dietary FDRM supplementation on productive performance of laying ducks are shown in Table 5. During the entire period, there were no significant effects (*p* > 0.05) by adding FDRM in laying ducks diet, regardless of the supplementation levels, on DEW, AEW, DEN, LR, ADFI, and F/E.

**Table 5:** Effects of FDRM on productive performance of laying duck^1^ (33–39 weeks of age).

Items^3^	Groups				SEM^2^	*p*-value
	F0	F1	F2	F3		
DEW (g/d)	788.20	799.09	792.87	795.93	11.74	0.990
AEW (g)	64.94	65.09	64.93	64.34	0.22	0.650
DEN [egg/(duck·d)]	0.76	0.77	0.76	0.77	0.01	0.977
LR (%)	75.85	76.79	76.36	77.36	1.14	0.977
ADFI [g/(duck·d)]	183.22	177.23	180.3	178.55	0.93	0.116
F/E (g/g)	3.74	3.57	3.67	3.61	0.05	0.706

^1^Data are the mean of eight replicates with 16 ducks each.

^2^SEM (standard error of the mean): the standard error of the average.

^3^DEW, daily egg weight = Gross egg weight laid in experimental period/49; AEW, average egg weight; DEN, daily egg number = Gross egg numbers laid in experimental period/49/16; LR, laying rate; ADFI, average daily feed intake; F/E, the ratio of feed to egg; FDRM, *Fagopyrum dibotrys* rhizoma meal; F0, control group; F1, the group supplemented with 1% FDRM; F2, the group supplemented with 2% FDRM; F3, the group supplemented with 3% FDRM.

### 3.2 Egg quality

The egg quality parameters of laying ducks are depicted in Table 6. Compared with the F0 group, the shell strength and yolk color in F2 and F3 groups were significantly increased (*p* < 0.05). Furthermore, the yolk color in F1 group and the shell proportion in F3 group were significantly (*p* < 0.05) higher than that in F0 group. No significant differences were observed in shape index, shell thickness, albumen height, Haugh unit, vitellus proportion, and albumen proportion (*p* > 0.05), in response to dietary FDRM supplementation levels.

**Table 6:** Effects of FDRM on egg quality of laying ducks^1^ (39 weeks of age).

Items^4^	Groups				SEM^2^	*p*-value^3^
	F0	F1	F2	F3		
Shape index (SI)	1.36	1.35	1.35	1.35	0.003	0.782
Shell strength (N/m^2^)	39.69^b^	43.12^ab^	43.88^a^	44.80^a^	0.61	0.006
Shell thickness (mm)	0.37	0.37	0.38	0.38	0.002	0.124
Albumen height (mm)	7.00	7.08	6.83	6.75	0.09	0.544
Haugh unit	80.74	81.59	80.20	79.32	0.64	0.674
Yolk color	5.52^b^	5.76^a^	5.77^a^	5.73^a^	0.03	0.003
Vitellus proportion (%)	31.55	31.54	31.74	31.60	0.13	0.953
Albumen proportion (%)	60.02	59.55	59.60	59.27	0.15	0.385
Shell proportion (%)	8.48^b^	8.91^ab^	8.83^ab^	9.13^a^	0.07	0.003

^1^Data are the mean of eight replicates with 16 ducks each.

^2^SEM (standard error of the mean): the standard error of the average.

^3^In the same row, values with no letter superscripts mean no significant difference (*p* > 0.05), while with different letter superscripts mean significant difference (*p* < 0.05).

^4^Shape index (SI) = egg length/egg width; FDRM, *Fagopyrum dibotrys* rhizoma meal; F0, control group; F1, the group supplemented with 1% FDRM; F2, the group supplemented with 2% FDRM; F3, the group supplemented with 3% FDRM.

### 3.3 Egg nutritional value

#### 3.3.1 Nutrient composition of egg

For the conventional nutrient levels of the vitellus in laying ducks, no significant dietary effects were observed across the measured parameters (moisture; crude protein; ether extract; cholesterol and Ca) (Table 7). Nevertheless, compared with the F0 group, the Ca content in F2 and F3 groups tend to improve as the inclusion level of FDRM increased (*p* = 0.073).

**Table 7:** Effects of FDRM on conventional nutrient levels in the vitellus of laying ducks (fresh matter basis)^1^.

Items	Groups				SEM^2^	*p*-value
	F0	F1	F2	F3		
Moisture (%)	49.57	49.75	49.25	49.61	0.10	0.410
Crude protein (%)	17.70	18.00	17.88	17.96	0.07	0.420
Ether extract (%)	10.61	10.53	10.52	10.38	0.06	0.646
Cholesterol (mg/g)	9.06	8.26	7.96	8.66	0.19	0.211
Ca (mg/g)	0.58	0.58	0.61	0.62	0.01	0.073

^1^Data are the mean of eight replicates with 16 ducks each.

^2^SEM (standard error of the mean): the standard error of the average.

FDRM, *Fagopyrum dibotrys* rhizoma meal; F0, control group; F1, the group supplemented with 1% FDRM; F2, the group supplemented with 2% FDRM; F3, the group supplemented with 3% FDRM.

#### 3.3.2 Amino acids composition

As can be seen from Table 3, the contents of Thr, Val, Ile, Leu, Asp., Glu, Gly, Cys, total EAAs, total AAs, and umami AAs in the vitellus showed significant response to the increasing FDRM supplement levels (*p* < 0.05). Compared with the control group (F0), there was no difference in the amino acids profile in F1 group (*p* > 0.05), dietary supplementation with 2% FDRM (F2) group could markedly increase the contents of Thr, Leu, Asp., Glu, Gly, Cys, total EAAs, total AAs, and umami AAs (*p* < 0.05), while the Thr and Asp concentrations in the group supplemented with 3% FDRM (F3) were statistically heighted (*p* < 0.05), with no dietary effect on other amino acids contents (*p* > 0.05). Furthermore, compared with the F1 group, the F2 group significantly increased the contents of Val, Ile, Leu, Gly, Cys, and total EAAs (*p* < 0.05), without significantly affecting the contents of other amino acids (*p* > 0.05).

#### 3.3.3 Fatty acids profile

Table 4 showed the effect of dietary FDRM addition on the fatty acids profile in the vitellus of laying ducks. The levels of C16:0 and total SFAs in the F1 and F2 groups exhibited a significant decrease compared to the F0 group (*p* < 0.05), while the UFAs:SFAs ratio increased significantly (*p* < 0.05). Moreover, the levels of C14:0 significantly decreased in the F2 and F3 groups (*p* < 0.05), the F3 group statistically heighted the contents of C20:1 and C20:3, markedly reduced the contents of total SFAs (*p* < 0.05). Compared with the F0 group, the PUFAs:SFAs ratio in the vitellus from the F1 group displayed a remarkable enhancement (*p* < 0.05).

### 3.4 Serum biochemical parameters

For serum biochemical parameters, the levels of TP and BUN along with the concentration of Ca in serum of laying ducks showed significant responses with increasing levels of FDRM in the diets (Table 8, *p* < 0.05). The serum levels of TP in F2 group and BUN in F3 group were significantly (*p* < 0.01) higher than that in F0 group. Additionally, in comparison to the F2 group, the F1 and F3 group dramatically lowered the Ca concentration in serum of laying ducks.

**Table 8:** Effects of FDRM on serum biochemical parameters of laying ducks^1^.

Items^4^	Groups				SEM^2^	*p*-value^3^
	F0	F1	F2	F3		
TP (g/L)	30.26^b^	31.88^ab^	35.20^a^	32.4^ab^	0.49	0.002
ALB (g/L)	15.91	15.67	17.34	15.88	0.24	0.107
BUN (mmol/L)	6.25^b^	5.63^b^	8.47^ab^	10.29^a^	0.54	0.005
AKP (KU/100 mL)	20.28	19.76	20.24	22.94	1.21	0.900
Ca (mmol/L)	1.34^ab^	1.29^b^	1.46^a^	1.29^b^	0.02	0.030
TG (mmol/L)	6.32	4.29	5.06	4.67	0.33	0.169
TC (mmol/L)	2.43	3.85	2.37	2.58	0.21	0.210
HDL (mmol/L)	1.12	1.85	1.19	1.50	0.11	0.059
LDL (mmol/L)	0.76	0.83	0.67	0.66	0.04	0.449

^1^Data are the mean of eight replicates with 16 ducks each.

^2^SEM (standard error of the mean): the standard error of the average.

^3^In the same row, values with no letter superscripts mean no significant difference (*p* > 0.05), while with different letter superscripts mean significant difference (*p* < 0.05).

^4^TP, total protein; ALB, albumin; BUN, blood urea nitrogen; AKP, alkaline phosphatase; Ca, calcium; TG, triglycerides; TC, total cholesterol; HDL, high-density lipoprotein; LDL, low-density lipoprotein; FDRM, *Fagopyrum dibotrys* rhizoma meal; F0, control group; F1; the group supplemented with 1% FDRM; F2, the group supplemented with 2% FDRM; F3, the group supplemented with 3% FDRM.

## 4 Discussion

This experiment aimed to investigate the effects of dietary supplementation with FDRM on laying performance, egg quality, egg nutritional value, and serum biochemical indicators of *Shanma* laying ducks. Our findings demonstrated that dietary inclusion of FDRM had no significant impact on daily egg weight, average egg weight, daily egg number, laying rate, average daily feed intake, and the ratio of feed to egg in laying ducks, but markedly enhanced shell strength and yolk coloration. Currently, there is limited research on the application of *F. dibotrys* in laying ducks. However, studies in laying hens (23) and broilers (30) are consistent with the results of this experiment, showing that *F. dibotrys* and its extracts have no significant impact on production performance. This also aligns with the findings of our previous research on reproductive performance (22). In general, compared to *Fagopyrum esculentum* (common buckwheat) and *Fagopyrum tataricum* (tartary buckwheat), *F. dibotrys* (golden buckwheat), as a member of the *Fagopyrum* genus, contains a greater variety of flavonoids and phenolics, exhibits stronger antimicrobial activity, and demonstrates intermediate antioxidant capacity (17, 31). Additionally, Chinese herbal medicine rich in flavonoids and phenolic acids have been shown to improve poultry health status and egg production performance (32, 33). Zhang et al. (26) found that supplementation of Mulberry leaf extract (rich in flavonoids such as rutin and phenolic acids like chlorogenic acid) in Lohmann Silber layers diet showed no adverse effects on production performance, yet significantly improved yolk pigmentation. In contrast, Chen et al. (3) and Feng et al. (34) reported that dietary supplementation with honeycomb extracts (rich in flavonoids such as quercetin and phenolic acids like caffeic acid) and *Eucommia ulmoides* leaf powder (rich in phenolic acids such as chlorogenic acid) in laying ducks have no significant improvements in laying performance and egg quality. Similarly, in the study by Iskender et al. (35), it was observed that no significant differences in laying performance and eggshell quality, in response to dietary supplementation with hesperidin, naringin and quercetin (All belong to the flavonoids). These discrepancies might be attributed to variations in Chinese herbal medicine types, poultry breeds and diets. Moreover, the intensified yolk pigmentation is likely attributable to the abundant bioactive constituents in *F. dibotrys*, notably flavonoids and phenolic acids, whose potent antioxidant activity helps preserve and deposit pigments by inhibiting the oxidation of carotenoids within the yolk (36). Given the limited existing research on FDRM in laying duck nutrition, further mechanistic investigations are warranted to elucidate its functional properties.

Duck eggs, containing abundant protein and amino acids, fatty acids, minerals and vitamins, serve as an excellent source of essential nutrients for human food and health. The primary indicators for assessing their nutritional value and sensory quality typically encompass amino acids composition and fatty acids profiles (3, 24). As fundamental building blocks of life, essential amino acids such as lysine, methionine, threonine, and phenylalanine not only play critical roles in regulating lipid and protein metabolism but also constitute indispensable nutrients that cannot be endogenously synthesized by animals and must be supplemented through dietary intake (37). This study revealed that compared to the control group, the 2% FDRM-supplemented group significantly increased the contents of total amino acids (by 9.27%), total essential amino acids (by 9.34%), and umami amino acids (by 7.62%) in egg yolks, confirming the beneficial effect of FDRM on the nutritional value of duck eggs. Currently, there is limited research on the application of *F. dibotrys* in laying ducks. Modern pharmacological studies have shown that golden buckwheat is rich in a variety of flavonoids and phenolic compounds, such as quercetin, rutin, gallic acid, and proanthocyanidins, which endow it with significant antioxidant properties (21, 31). The potential underlying mechanisms may involve enhancing antioxidant capacity and modulating the expressions of genes related to amino acid metabolism (38). Notably, Yao et al. (39) also reported that sea buckthorn extract rich in flavonoid such as quercetin significantly improved the contents of total amino acids, essential amino acids, and umami amino acids in eggs through a similar mechanism. Nevertheless, current research remains insufficient in identifying the specific bioactive components within FDRM and their molecular targets, which represents a critical focus for future investigations.

Accumulating evidence highlights the dual implications of fatty acids intake on human health. Scientific evidence indicated that high intake of saturated fatty acids is associated with elevated risks of type 2 diabetes and cardiovascular disorders, while monounsaturated and polyunsaturated fatty acids demonstrate various protective health effects, including anti-inflammatory effects, regulation of glucose and lipid metabolism, and promotion of muscle growth (28, 40). In the present trial, adding FDRM in laying duck diet led to an increase in the ratio of unsaturated to saturated fatty acids (UFAs:SFAs) and a decrease in total SFAs in egg yolks, with the 2% FDRM group showing a pronounced decrease in total SFAs. Researches conducted by Zhang et al. (23) and Zhang et al. (24) revealed that supplementing laying hen diets with *F. dibotrys* stems and leaves or their extracts could improve the amino acids composition and content of whole eggs while significantly increasing the levels of C20:4 and C22:6 in egg yolks. Furthermore, Chen et al. (3) also reported that the contents of total unsaturated fatty acids, monounsaturated fatty acids, and polyunsaturated fatty acids in duck eggs showed an increasing trend with the dietary supplementation level of honeycomb extracts, while the total saturated fatty acids content decreased significantly. Our findings are in full accordance with these previously established conclusions. The potential mechanism is that the polyphenols in FDRM scavenge ROS, protecting PUFAs from oxidative degradation and thereby reducing lipid peroxidation (41). In addition, the flavonoids in *F. dibotrys* enhance fatty acids elongation, thereby increasing the deposition of PUFAs in egg yolks (34).

Serum biochemical parameters serve as critical indicators for assessing metabolic status and health conditions in animals, primarily encompassing serum enzymes, protein, and lipid metabolites. Serum TP, composed of ALB and GLB, reflects protein absorption and metabolism in the body. Elevated TP levels indicate enhanced protein metabolism and immune competence (42–44). The experimental data confirmed that FDRM supplementation led to a marked rise in serum TP concentration, aligning with findings reported by Chen et al. (43) and Zhang et al. (24). Serum BUN levels serve as an indicator of protein and amino acids utilization, with decreased concentrations suggesting favorable amino acids balance (45). Tan et al. (44) discovered that dietary supplementation with 1% *F. dibotrys* in broilers decreased the serum BUN level. However, this study revealed that the 3% FDRM supplementation group significantly elevated the serum BUN content compared to the control group, diverging from the aforementioned findings. This discrepancy suggested that dietary FDRM supplementation should not exceed 3%, as higher levels may compromise protein utilization efficiency.

## 5 Conclusion

This study found that adding 2% FDRM to the diet of *Shanma* laying ducks could improve the shell strength, yolk color, and shell thickness in duck egg. Additionally, it improved the fatty acids profile, increased the levels of total amino acids, essential amino acids and umami amino acids in egg yolks. Concurrently, elevated the serum total protein levels indicated augmented physiological processes related to protein synthesis. These modifications suggested that 2% FDRM had a potential improvement in egg quality and egg nutritional value, with no negative impact on laying performance and health status of *Shanma* laying ducks. Under the conditions of this experiment, FDRM could be effectively utilized as a phytogenic feed additive in *Shanma* laying duck diets.

## Data Availability

The original contributions presented in the study are included in the article/supplementary material, further inquiries can be directed to the corresponding authors.
